# Mitochondrial tRNA-Derived Fragments and Their Contribution to Gene Expression Regulation

**DOI:** 10.3389/fphys.2021.729452

**Published:** 2021-09-03

**Authors:** Athanasios-Nasir Shaukat, Eleni G. Kaliatsi, Vassiliki Stamatopoulou, Constantinos Stathopoulos

**Affiliations:** Department of Biochemistry, School of Medicine, University of Patras, Patras, Greece

**Keywords:** mitochondrial tRNA-derived fragments, mitochondrial tRNAs, tRNA-derived fragments, ncRNAs, mitochondria

## Abstract

Mutations in human mitochondrial tRNAs (mt-tRNAs) are responsible for several and sometimes severe clinical phenotypes, classified among mitochondrial diseases. In addition, post-transcriptional modifications of mt-tRNAs in correlation with several stress signals can affect their stability similarly to what has been described for their nuclear-encoded counterparts. Many of the perturbations related to either point mutations or aberrant modifications of mt-tRNAs can lead to specific cleavage and the production of mitochondrial tRNA-derived fragments (mt-tRFs). Although mt-tRFs have been detected in several studies, the exact biogenesis steps and biological role remain, to a great extent, unexplored. Several mt-tRFs are produced because of the excessive oxidative stress which predominantly affects mitochondrial DNA integrity. In addition, mt-tRFs have been detected in various diseases with possible detrimental consequences, but also their production may represent a response mechanism to external stimuli, including infections from pathogens. Finally, specific point mutations on mt-tRNAs have been reported to impact the pool of the produced mt-tRFs and there is growing evidence suggesting that mt-tRFs can be exported and act in the cytoplasm. In this review, we summarize current knowledge on mitochondrial tRNA-deriving fragments and their possible contribution to gene expression regulation.

## Introduction

Mitochondria have evolved as the powerhouse of cells and the central nexus for major metabolic pathways that utilize oxygen for ATP production in eukaryotic cells. As such, they represent a major hub of the intracellular communication that define the cell’s fate. Mitochondria-related networking and the bidirectional pathways that rule it have long been identified to have profound effects on cell’s metabolism, bioenergetics, and homeostasis. However, several details regarding the molecular mechanisms and gene networks involved still remain unknown. Human mitochondrial DNA exists in multiple copies in each organelle and consists of 37 genes including 2 mt-rRNAs, 22 mutations in human mitochondrial tRNAs (mt-tRNAs), and 13 coding genes which are translated through specific decoding by mitochondrial ribosomes to the essential subunits of respiratory complexes (Anderson et al., 1981). The expression of the mitochondrial genes provides, among others, energy supply and antioxidant defense depending also on several sets of devoted nuclear-encoded genes that act either in the mitochondrial milieu or at the mitochondrial membrane. Nuclear-encoded non-coding RNAs are also known to indirectly affect mitochondrial function either through regulating the expression and post-translational modifications of the approximately 1,500 proteins targeted to mitochondria or through their direct import and interaction with proteins and RNAs inside mitochondria (Gusic and Prokisch, 2020). Several miRNAs or long non-coding RNAs, like *SAMMSON*, modulate mitochondrial function by affecting the import of proteins (Leucci et al., 2016; Vendramin et al., 2017; Jeandard et al., 2019; Kaliatsi et al., 2020). It should be noted that in human, although RNA import mechanisms into mitochondria have been proposed and involve PNPase (polynucleotide phosphorylase) activity located in the intermembrane space (reviewed in Vendramin et al., 2017 and Jeandard et al., 2019), an export mechanism is still elusive.

Current knowledge suggests that several perturbations in the regulation of the mitochondrial gene expression can lead to dysfunction of mitochondria. In turn, defective mitochondria are characterized by impaired energy production and depending on the broadness of the mutations, can lead to disorders with severe clinical symptoms that mainly affect the highly energy-demanding tissues, like brain, heart, and muscles. More specifically, mitochondrial DNA mutations, especially on mt-tRNAs, have been linked initially to syndromes like MERRF (myoclonic epilepsy with ragged red fibers), MELAS (mitochondrial encephalomyopathy, lactic acidosis, and stroke-like episodes) and cardiomyopathies (Gorman et al., 2016), and nowadays to the emergence of specific types of cancer (Stewart et al., 2015). The fact that many of the known mitochondrial diseases have their molecular basis on mutations in mt-tRNA genes indicates the central role of mitochondrial translation in the pathophysiology of the cells and the tissues. Point mutations in mt-tRNAs can affect the efficiency of the 5' and 3' processing, the epigenetic changes deriving from specific post-transcriptional modifications, the accuracy of tRNA aminoacylation and decoding during translation, and overall the mt-tRNA stability, resulting in many cases in severe inherited mitochondrial diseases (Yan et al., 2006; Zifa et al., 2007; Xue et al., 2019; Chujo and Tomizawa, 2021; Ji et al., 2021; Karasik et al., 2021). It should be noted that several mitochondrial tRNAs appear truncated in their mature form when compared to their cytosolic counterparts and in the case of tRNA^Ser(AGY)^, the D-stem and loop are missing. In addition, different point mutations in the same mt-tRNA molecule can result in different diseases, like in the case of mt-tRNA^Glu^ where specific mutations have been linked with maternally inherited diabetes and deafness, while others in the same gene with infantile transient mitochondrial myopathy (Webb et al., 2020). Moreover, mutations in mt-tRNAs can co-exist with mutations in genes encoding mitochondrial enzymes, an observation that highlights the phenotypic diversity among mitochondrial mutations with possible clinical significance (Zifa et al., 2008). The accurate transcription and maturation of mt-tRNAs are essential to successfully decode several codon deviations from the conventional universal genetic code, like the UGA stop codon which encodes for tryptophan, the isoleucine AUA codon which encodes for methionine, and the two arginine codons AGA and AGG which in mitochondria designate termination (Kummer and Ban, 2021). As a result, defects that affect the mt-tRNA stability and utilization as substrate can impact not only the mitochondrial translation rate but also the pool of available molecules leading to specific tRNA fragmentation that could affect the mitochondrial gene expression and potentially the crosstalk with the cytoplasm and the nucleus.

Although fragments of tRNAs were sporadically observed many years ago, the recent advance of sequencing technologies and bioinformatics tools brought tRNA-derived fragments (tRFs) to the spotlight, categorizing them in groups depending on their site of origin on tRNA molecules (Kumar et al., 2016). More specifically, tRFs are classified as tRF-1s, tRF-5s, tRF-3s, 5'-tRNA halves, 3' tRNA halves, or itRFs (internal tRFs) and are produced depending also on the external stimulus. More interestingly, each distinct class has been linked to specific biological function; global translation inhibition has been attributed mainly to 5' tRNA halves (but also to tRF-5s), translation repression of specific mRNAs mainly to tRF-3s, inhibition of specific gene transcription to tRF-5s, inhibition of viral RNA translation to tRF-1s, inhibition of apoptosis to 5' tRNA halves, and regulation of retrotransposon expression both to tRF-3s and tRF-5s (Schimmel, 2018; Su et al., 2020). In general, although tRFs production can be induced by stress, or the lack of specific post-transcriptional tRNA modifications, a certain number of tRFs has been proposed to act like miRNAs and to mediate gene silencing (Kumar et al., 2014; Skeparnias et al., 2020). Finally, although several known ribonucleases seem to contribute in the production of specific tRFs (see below), it is also likely that additional ribonucleolytic activities and/or biogenesis steps may mediate tRFs biogenesis (Su et al., 2020).

The advance of high throughput methodologies, such as next-generation sequencing and meta-analysis of existing data, has lately revealed the existence of mitochondrial tRNA-derived fragments (mt-tRFs) with possible correlation to pathological phenotypes. Strikingly, mt-tRFs appear as contributors to the synchronization of a series of essential cellular and mitochondrial biological processes, acting as “couriers” not only between nucleus and mitochondrion, but also between different cells of the same or different species (Saikia et al., 2014; Meseguer, 2021). Herein, the putative production pathways of mt-tRFs and their effect on gene expression regulation, along with the role of tRNA processing factors that may contribute to differential mt-tRF expression profiles in pathogenic phenotypes, are summarized.

## Current Knowledge on Mt-tRNA-Derived Fragments Biogenesis

Nowadays, the powerful methodological tools can provide an unprecedented opportunity to approach the nuclear-mitochondrial communication and the delicate coordination of nuclear- and mitochondrial-encoded factors, enzymes, and cofactors aiming to delineate the mechanisms that drive to specific mitochondrial diseases. Although at the beginning, tRFs were first considered tRNA degradation byproducts, later studies showed they are abundant in all species and are generated following a specific endonucleolytic cleavage of precursor or mature tRNAs (Grafanaki et al., 2019; Su et al., 2020). Today, tRFs represent important modulators of various processes, including translation, apoptosis, anti-viral defense, and response to nutrient starvation or oxidative stress. Interestingly, with the accumulation of sequencing data and the development of highly specialized bioinformatic platforms, it becomes evident that not only nuclear-encoded tRNAs but also mt-tRNAs can produce tRFs (Kumar et al., 2015; Zheng et al., 2016; Pliatsika et al., 2018; Looney et al., 2020). Of note, differential expression of mt-tRFs has been observed in cancer, as a result of mtDNA mutations and as a response to external stimuli, including infections from pathogens (Meseguer et al., 2019; Nätt et al., 2019; Karousi et al., 2020; Looney et al., 2020). Subsequently, mt-tRFs appear to actively participate in the intracellular communication and the mitochondrial pathophysiology, affecting also the cytoplasmic translation machinery (Loher et al., 2017; Meseguer et al., 2019; Looney et al., 2020). Finally, there is growing interest regarding the possible transportation of mt-tRFs across the mitochondrial membrane which raises further questions on which and how many ribonucleases and modifiers could be implicated to mt-tRF biogenesis.

**Figure 1:**
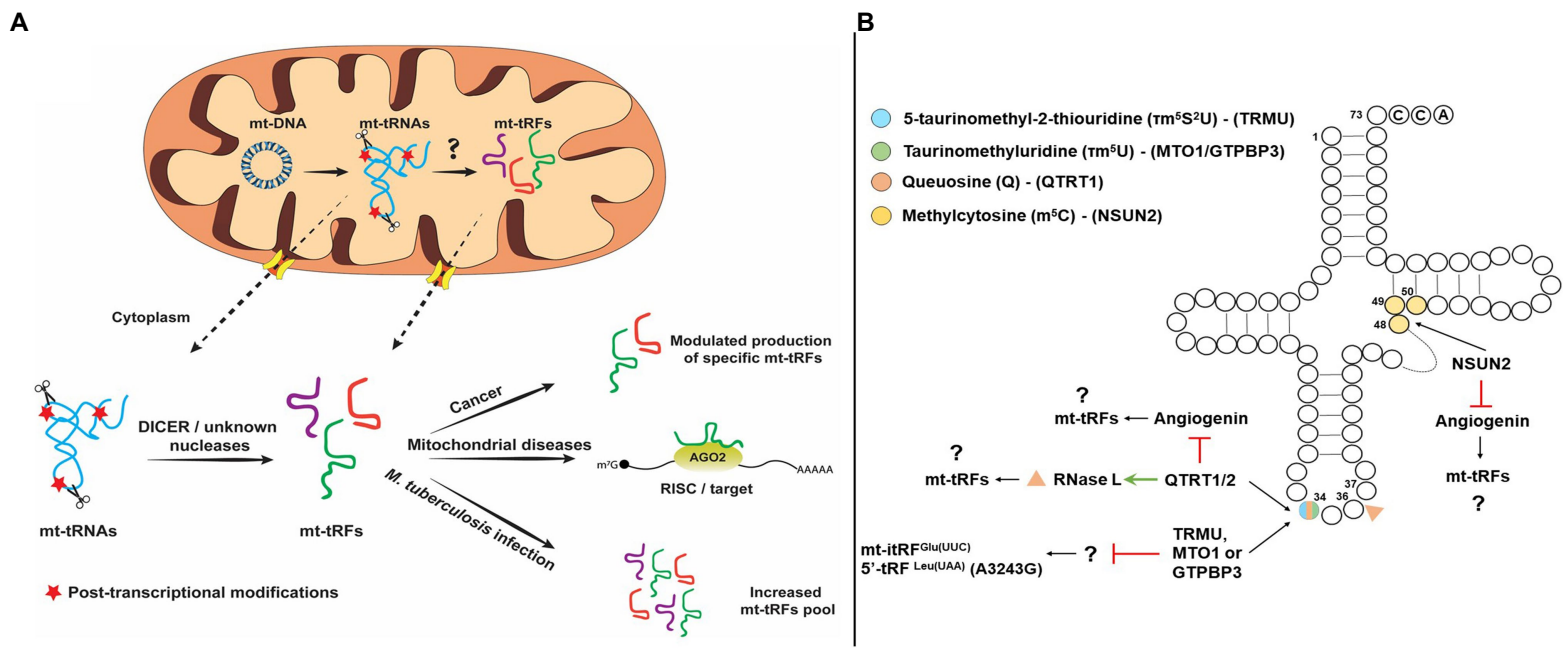
**(Α)** Schematic representation of putative pathways of mitochondrial tRNA-derived fragments (mt-tRFs) biogenesis and biological function and (**Β)** Hypothetic model of mt-tRNA cleavage regulation by post-transcriptional modifications.

So far, specific endoribonucleases have been identified that participate in tRF production. The first endoribonucleases identified were RNase Z^L^/ELAC2 and Angiogenin (ANG). RNase Z^L^/ELAC2 is responsible for the maturation of the 3' end of tRNAs through pre-tRNA cleavage downstream of the discriminator base and the only activity which is localized both in the nucleus and mitochondria. ANG, on the other hand, cleaves predominantly in the anticodon loop and is mainly responsible for stress-induced tRFs production (Lee et al., 2009; Yamasaki et al., 2009; Siira et al., 2018). Additional enzymes that have been identified to participate in the generation of tRFs include SLFN11 (Schlafen 11), SLFN13, and RNase L (Li et al., 2012, 2018; Donovan et al., 2017; Yang et al., 2018), but interestingly, none of these activities have been detected in mitochondria. Although, the cleavage site of SLFN11 is unknown, SLFN13 cleaves in the junction of the T and anticodon stems of tRNAs, while RNase L cleaves at the anticodon loop (Donovan et al., 2017; Li et al., 2018; Yang et al., 2018). Dicer is also implicated to the generation of tRFs but only in specific cases (Hasler et al., 2016; Kuscu et al., 2018). For example, precursor tRNAs that escape the La-dependent maturation pathway are folded into structures that are recognized by exportin 5 (Xpo5) as miRNA precursors and exported in the cytoplasm, where they are cleaved by DICER producing tRFs, which are then loaded to AGO proteins (Hasler et al., 2016). Aside from La that protects tRNAs from being cleaved, it was found recently that SLFN2 binds mature tRNAs upon T-cell activation and inhibits oxidative stress-induced tRF biogenesis by ANG, thus preventing global translation inhibition (Yue et al., 2021). However, in most cases, the mechanism for producing tRNA halves, tRF-5s, tRF-3s, and itRFs is still elusive and the question whether specific tRNA-halves production is tightly regulated remains open (Su et al., 2019, 2020). Given the current knowledge regarding the activities that mediate tRFs production from nuclear-encoded tRNAs, the respective activities that are responsible for mt-tRFs production are still unknown (Figure 1A). It should also be noted that it remains debatable whether and which mt-tRNAs are processed either in the mitochondrion or the cytoplasm to generate mt-tRFs. Enzymes that are targeted into mitochondria could affect mt-tRF biogenesis, like RNase Z^L^/ELAC2, which has been identified as a hotspot for mutations linked with mitochondrial diseases, such as hypertrophic cardiomyopathy but also with prostate cancer (Siira et al., 2018; Saoura et al., 2019). Although most of these mutations impair the efficient maturation of the 3' end of both the nuclear and mitochondrial tRNAs, the effect in mitochondria is more intense. For example, the R781H substitution in RNase Z^L^/ELAC2, which has been linked with prostate cancer and hypertrophic cardiomyopathy, mainly affects mt-tRNA processing while has no major differences on the nuclear pre-tRNA processing (Minagawa et al., 2005; Saoura et al., 2019). This effect could potentially lead to production of differential mt-tRFs levels and a broader effect in gene expression regulation, both inside and outside mitochondria. Another candidate involved in the mt-tRF generation is LACTB2 (Lactamase B2), a mitochondrially targeted ribonuclease that is able to cleave single-stranded RNAs *in vitro* (Levy et al., 2016). Although LACTB2 is mainly involved in mt-mRNA turnover, *in vitro* cleavage assays demonstrated that in the presence of a hairpin-structured RNA substrate, LACTB2 cleaves only in the loop region and may also target the T, D, or anticodon loops of mt-tRNAs, generating mt-tRFs. Given the putative existence that a mechanism that actively shuffles mitochondrial RNAs across the mitochondrial membrane to be processed in the cytoplasm by known endoribonucleases, the possibility of mt-tRFs production by cytoplasmic enzymes cannot be excluded (Bruni et al., 2017; Jády et al., 2018; Figure 1A). Interestingly, it was shown recently that silencing of either *DICER* or *AGO2* downregulated the expression of mt-itRF^Glu(UUC)^, mt-5'tRF^Leu(UUA)^ carrying the A3243G mutation and mt-3'tRF^Val(UAC)^ (Meseguer et al., 2019). This observation suggests that DICER could potentially cleave mt-tRNAs as well, however, whether this occurs in the cytoplasm or inside mitochondria is unclear (Figure 1A; Meseguer, 2021). The reason why *AGO2* silencing leads to decreased expression of mt-tRFs is also not clear. Considering the possibility that AGO2-bound mt-tRFs exhibit increased half-lives, the absence of AGO2 could increase the susceptibility of mt-tRFs to degradation. Although the defined mitochondrial export mechanism is still elusive, it is apparent that apoptosis and autophagic turnover of mitochondria cause mt-tRNAs to translocate in the cytoplasm (Marnef et al., 2016; Jády et al., 2018). Under these conditions, several RNA-binding proteins, including AGO2, Y-box-binding proteins (YBX1 and YBX3), mRNA-binding proteins (SRSF1, SRSF2, SRSF3, hnRNP A1, and hnRNP H), and polypyrimidine-binding proteins, have been found to interact with a subset of mt-tRNAs in the cytoplasm. This interaction may indicate a possible mechanism *via* which, specific mt-tRNAs are processed by cytoplasmic ribonucleases, like ANG and DICER to produce mt-tRFs (Maniataki and Mourelatos, 2005; Marnef et al., 2016; Jády et al., 2018). However, as discussed above, additional ribonucleases may also be involved in cytosolic mt-tRF biogenesis.

Apart from the previously mentioned mechanisms, specific modification patterns on mt-tRNAs are key factors and not only contribute to structural stability and recognition by enzymes and proteins, but also act protectively against aberrant cleavage and fragmentation. More interestingly, lack of specific modifications has been linked with the production of specific tRFs under different conditions. Queuosine (Q) is an important modification of G34 of GUN anticodons and responsible for the efficient decoding of NAY codons in the cytosol and mitochondria (where N corresponds to any base and Y to pyrimidine) and lack of this modification affects the cleavage of both RNase L and ANG (Suzuki, 2021). More specifically, RNase L cleaves the *in vitro*-transcribed tRNA^His^ in three different sites; however, isolation of endogenous and modified with Q34 tRNA^His^ is only cleaved at position 36 (Donovan et al., 2017). On the other hand, Q34 inhibits ANG cleavage altogether (Wang et al., 2018). Thus, considering that Q34 modification status besides the decoding fidelity and efficiency affects also the cleavage specificity of mt-tRNAs and it may modulate mt-tRF production. Interestingly, the absence of an identified biosynthetic pathway for the micronutrient queuine in human, which is required for queuosine formation, suggests that the microbiome is mainly responsible for the existence of this modification (Müller et al., 2019). This observation provides a possible link between the role of the microbiome in tRF biogenesis. In the same line, a recent report that analyzed in detail the human tRNA modification landscape underlined the fact that several modifications occur near sites that are susceptible to potential pathogenic mutations. The presence of non-canonical nucleotides near modification sites could affect the binding affinity of modifying enzymes and/or affect their enzymatic efficiency, resulting in hypomodified and unstable mt-tRNAs, which are unable to participate efficiently in mitochondrial translation and are prone to cleavage (Suzuki et al., 2020). Fruit flies and mice lacking NSUN2 (NOP2/Sun methyltransferase 2) or DNMT2 (DNA methyltransferase 2), the methyltransferases that modify cytosines at position 5 (m^5^C), were found to bear increased levels of stress-induced tRFs that are produced by ANG (Figure 1B). The correlation of NSUN2 with other modification enzymes that modify mt-tRNAs regulates their stability and functional properties (Suzuki, 2021). NSUN2 has recently been detected in mitochondria and is responsible for introduction of m^5^ at C48, C49, or C50 of mt-tRNAs (Shinoda et al., 2019; Van Haute et al., 2019). Taking into account that *NSUN2* expression is dynamically modulated in various physiological conditions, such as cancer, neurodegenerative diseases, and during differentiation, it is possible that differential methyltransferase activity in mitochondria may also drives the production of specific mt-tRFs (Blanco et al., 2014; Chellamuthu and Gray, 2020). Of note, queuosine hypomodification has been reported in several cancers and in multiple sclerosis *via* reduced activity of tRNA guanine transglycosylase (TRT) or *via* promoter methylation of the genes coding for the TRT subunits (*QTRT1* and *QTRT2*) (Baranowski et al., 1994; Pathak et al., 2005; Varghese et al., 2017; Hayes et al., 2020).

Several tRNA modifications that are mitochondria specific-like 5-taurinomethyluridine (τm^5^U), 5-taurinomethyl-2-thiouridine (τm^5^S^2^U), and 5-formylcytidine (f5C) that could play an important role in the regulation of mt-tRNA cleavage. Silencing of either *TRMU*, *GTPBP3,* or *MTO1* genes that code for enzymes that modify U34 of mt-tRNAs led to increased expression of mt-itRF^Glu(UUC)^, indicating that the modification state of mt-tRNAs can modulate mt-tRF biogenesis (Figure 1B; Schaefer et al., 2010; Tuorto et al., 2012; Meseguer et al., 2019). Also, the mt-tRNA^Leu(UUR)^ harboring the MELAS mutation A3243G which is known to prevent the τm^5^ modification of U34, also leads to the upregulation of a mt-tRF-5 derived from mt-tRNA^Leu(UUR)^. Based on these observations, it has been proposed that lack of τm^5^U34 could drive the generation of specific mt-tRFs (Meseguer et al., 2019). Finally, τm^5^U frequency in cultured cell lines and in animal models is highly affected by dietary uptake of taurine (Asano et al., 2018). Upon taurine starvation in cultured cells and animal tissues, τm^5^U is replaced by cmnm^5^U (5-carboxymethylaminomethyluridine), which could potentially alter the cleavage patterns of mt-tRNAs by specific ribonucleases, leading to the production of differential mt-tRF profiles.

## Mitochondrial tRNA-Derived Fragments in Pathophysiology

The role of tRFs in gene expression regulation has gradually drawn the interest, with specific tRFs deriving from nuclear-encoded tRNA species to be considered as novel diagnostic and prognostic biomarkers of several types of disorders, such as cancer, as well as possible druggable targets for cancer treatment (Srinivasan et al., 2019; Zhu et al., 2019). Although, the biological function of mt-tRFs has not been clearly defined, they could bear similar value (Table 1). A previous extensive analysis of sRNA-seq data from mitoplasts derived from 143B osteosarcoma cells revealed the existence of several sRNA (small RNA) species that map to mitochondrial tRNAs (Mercer et al., 2011). Interestingly, most of the mt-tRFs detected in that study correspond to 5' mt-tRFs but also to the region downstream of tRNA 3' cleavage side, which correspond to tRF-1s (Mercer et al., 2011). Moreover, the 5' mt-tRFs harbored several sequencing mismatches at positions known for being modified, indicating their origin from mature mt-tRNAs. Several studies have attempted to correlate the differential mt-tRFs patterns with various pathophysiological conditions. A reported correlation between differential abundance and length distribution of mt-tRFs between datasets from breast invasive carcinoma (BRCA; derived from The Cancer Genome Atlas project) and patient-derived lymphoblastoid cell lines (LCL) highlighted the importance of mt-tRFs patterns as biomarkers (Telonis et al., 2015). It is likely that specific mt-tRNAs produce only a specific type of mt-tRF (i.e., either 5'-tRF or 3'-tRF or i-tRF) while others can be fragmented to all types. Moreover, LCL and BRCA datasets contained not only different mt-tRF pools, but also different types of mt-tRFs generated from the same type of mt-tRNA, suggesting that the type of mt-tRF produced by each individual mt-tRNA is cancer type specific. A recent report interconnected the low overall survival of patients suffering from chronic lymphocytic leukemia (CLL) with the presence of an internal mt-tRF (Karousi et al., 2020). In this study, non-coding RNAs were quantified *via* bioinformatic analysis in CLL patients. Interestingly, a novel mt-tRF was identified derived from the mt-tRNA^Phe^ bearing the anticodon GAA and termed i-tRF^Phe(GAA)^. Specifically, i-tRF^Phe(GAA)^ could serve as a potential prognostic biomarker in CLL, since its high expression level was linked with poor outcome of the disease (Karousi et al., 2020). Although these observations await experimental validation and more in-depth investigation to attribute its biological significance, they illustrate the dynamic nature of mt-tRF biogenesis, which may reflect to their distinct biological functions under different conditions or pathophysiologies. Moreover, apart from the apparent correlation of mt-tRFs with several cancer types, their role has been also emerged in infections from pathogens. Although most studies have focused on viral infections, it was recently reported that infection with *Listeria monocytogenes* and *Mycobacterium tuberculosis* affected the tRF production in macrophages and more importantly, infection by *M. tuberculosis* significantly affected the abundance of the mt-tRFs pool (Looney et al., 2020). The study suggested that this effect could be driven by the hypoxic conditions that usually accompany *M. tuberculosis* infections which, in turn, alter the mitochondrial physiology and subsequently the mt-tRF production.

**Table 1:** Examples of mitochondrial tRNA fragments affected under various conditions.

mt-tRF	Physiological State	Observation	Biological function	Reference
i-tRF^Phe(GAA)^	Chronic lymphocytic leukemia (CLL)	Increased levels in PBMCs correlated with lower survival	Unknown	Karousi et al., 2020
Various mt-tRFs	Infection with intracellular pathogens, mainly *Mycobacterium tuberculosis*	Deregulation of expression	Unknown	Looney et al., 2020
i-tRF^His(GTG)^, 5' and i-tRF^Ser(TGA)^, 3'-half/3'-tRF/i-tRF^Thr(TGT)^, 5' and 3'-tRF^Val(TAC)^	Sugar-rich diet	Increased abundance in spermatozoa and increased motility	Unknown	Nätt et al., 2019
itRF^Glu(UUC)^	MELAS	Upregulation in MELAS cybrid cells	Targets *MPC1* mRNA	Meseguer et al., 2019
5'-tRF^Leu(UAA)^ (A3243G), 3'-tRF^Val(UAC)^	MELAS	Upregulation in MELAS cybrid cells	Unknown	Meseguer et al., 2019
5'-tRF^Leu(UAA)^	MELAS	Downregulation in MELAS cybrid cells	Unknown	Meseguer et al., 2019

Very recently, the role of diet as factor that induces mt-tRFs production was described in relation to human sperm motility. Human sperm responds rapidly to changes in a sugar-rich diet exhibiting increased motility and upregulation of specific nuclear and mt-tRFs. Strikingly, although both nuclear and mt-tRFs production are sugar sensitive, the high percentage of mt-tRFs could correlate to the improved sperm motility (Nätt et al., 2019). In addition, considering that the modification state of tRNAs can affect the tRFs biogenesis, it seems that the mt-tRNA epitranscriptome can also give rise to the mt-tRFome (Martínez-Zamora et al., 2015). For example, the prevalent mutation A3243G, which leads to the MELAS disease, can cause differential expression of several mt-tRFs. This mutation resides inside the mt-tRNA^Leu(UUR)^ and prevents the writing of the τm^5^U34 modification that stabilizes the U-G wobble pair and makes the mutated mt-tRNA unable to decode the UUG codon efficiently (Kirino et al., 2004). Notably, a 5' mt-tRF derived from the mutated mt-tRNA^Leu(UUR)^ was upregulated in MELAS cybrid cells, indicating that the loss of τm^5^ modification at U34 favors the production of this mt-tRF. Finally, specific mt-tRFs and particularly, mt-itRF^Glu(UUC)^, mt-5'tRF^Leu(UUA)^ carrying the A3243G mutation and another mt-5'tRF^Leu(UAA)^, apart from being accumulated inside the mitochondria, have been detected also in the cytosol.

Similar to their cytoplasmic counterparts, mt-tRFs presumably can act like miRNAs to induce gene silencing and, in some cases, may share common mRNA targets (Meseguer et al., 2019; Skeparnias et al., 2020). Interestingly, the differential expressed mt-tRFs in MELAS cybrid cells are predicted to target several mRNAs involved in the regulation of striated and cardiac muscle contraction but also in the development of these tissues (Meseguer et al., 2019). Since myopathy and muscle spasms are common symptoms in MELAS, these mt-tRFs may facilitate disease progression by affecting muscle tissue function, on top of the mitochondrial dysfunction that results from reduced mitochondrial translation. In this context, a mt-itRF derived from mt-tRNA^Glu(UUC)^ was found upregulated in MELAS cybrid cells. This specific tRF directly interacts to and downregulates *MPC1* mRNA which encodes for the mitochondrial pyruvate carrier protein, leading to extracellular accumulation of lactate. Finally, mt-tRNA^Ser(UCN)^ is a hotspot for mutations (T7510C, T7511C, and A7445G) that can lead to non-syndromic hearing loss, meaning that hearing impairment is the main, and usually the only symptom. It is known that inner-ear cells have structurally unique mitochondria which may be why this is usually the main symptom in individuals carrying this mtDNA mutation (Lesus et al., 2019). The pathogenesis of these mutations has been attributed to the reduced steady state levels of mt-tRNA^Ser(UCN)^ because RNase Z^L^/ELAC2 cannot process these transcripts efficiently (Yan et al., 2006). One possibility that should be further explored is whether these mutations could result in the production of specific mt-tRFs deriving from mt-tRNA^Ser(UCN)^ and whether can be related to the progression of non-syndromic hearing loss.

Collectively, current knowledge suggests that among the main factors that lead to detection of differential levels of mt-tRFs in cancer includes, among others, rewiring of mitochondrial metabolism through translation deregulation. The latter indicates that several types of mitochondrial dysfunction, such as loss of mitochondrial membrane potential and mitochondrial calcium overload, can affect mt-tRF production, *via* their impact on mtDNA transcription and translation and possibly *via* impaired efficiency of non-coding RNAs and proteins import. Aberrant translation could increase the availability of mt-tRNAs for cleavage thus leading to production of mt-tRFs. On the other hand, reduced mtDNA transcription could lead to a reduction of the mt-tRNA pool and subsequently to overall availability for mt-tRFs production. Moreover, alterations during import of important proteins that are targeted into mitochondria can also potentially lead to differential mt-tRFs levels and the modulation of the genes that are potential targets.

## Concluding Remarks

Mitochondria, although rather simple organelles, facilitate essential metabolic cascades and sense environmental cues to regulate energy production, manage oxidative stress, and serve an overall normal cellular function. Due to the dynamic crosstalk with other subcellular compartments and their profound effects on pathophysiology, mitochondria represent a unique field for studying several regulatory circuits that affect gene expression. During recent years, tRFs have been established as a novel class of small non-coding RNAs that predominantly have protective effect but can also play role in the deregulation of translation. Based on the central role of mitochondrial tRNAs during mitochondrial translation, the corresponding mt-tRFs could modulate gene expression regulation in a similar way to achieve fine-tuning of the communication with the nucleus.

It must be underlined that almost half the mutations in mtDNA occur on tRNA genes with various effects on mt-tRNA biogenesis, aminoacylation efficiency, and mRNA decoding (Zifa et al., 2007; Yarham et al., 2010; Suzuki, 2021). The increased instability of such mutated mt-tRNAs as well as of mt-tRNAs bearing aberrant modifications could be the leading cause for the differential mt-tRF production related to disease onset or progression. In addition to known point mutations on mt-tRNA genes or to deficiencies of ribonucleases and modifiers, external stimuli spanning from bacterial infections to specific nutrients can affect the homeostasis of mitochondria and therefore can directly affect mt-tRFs production. For instance, taurine administration has been shown to ameliorate stroke episodes in MELAS patients and is now used as a treatment regimen in Japan (Rikimaru et al., 2012; Ohsawa et al., 2019; Suzuki, 2021). Therefore, it is worth studying the effect of taurine intake on mt-tRF production in afflicted tissues of MELAS patients. It is evident that exploring the mt-tRF biogenesis and post-transcriptional modifications of both nuclear and mitochondrial tRNAs consist a critical step to unlock new RNA-mediated nuclear-mitochondrial circuits implicated in the cell metabolism and pathological conditions.

Apart from the undisputed existence of mt-tRFs and their correlation to specific human disorders, several questions regarding their biogenesis and role remain open. It is important to decipher how and which mt-tRNAs or mt-tRFs are exported from mitochondria but also in which subcellular compartment production of the majority of mt-tRF occurs. Import and export through the mitochondrial membrane currently remain a mystery. For this reason, experimental tools, such as specific RNA-aptamers, antisense oligonucleotides, or even delivery of free RNA, could be developed to carefully and reliably determine mt-tRFs spatiotemporal localization (Dovydenko et al., 2021). The putative mechanisms which control import and export of mt-tRNAs and mt-tRFs could be targeted to treat mitochondrial diseases or to ameliorate secondary effects. The identification of specific enzymatic activities that are involved in mt-tRF production will provide valuable knowledge and will allow the better understanding of the possible role of mt-tRFs during intracellular communication. Given that the majority of mt-tRFs is accumulated inside mitochondria, it should be delineated which ribonucleases get involved, whether they are transported inside mitochondria and which is the cleavage pattern of their mt-tRNA substrates. Finally, considering that mitochondria originated from endosymbiotic events with prokaryotes, and regarding the similarities between mitochondria and bacteria, ranging from genome organization, membrane structure, and protein synthesis to overall structure, elucidation of the mechanism of mt-tRF biogenesis could provide new knowledge on the potential roles and biogenesis pathways of bacterial tRFs and *vice versa*.

## Author Contributions

A-NS, EK, VS, and CS contributed to the manuscript writing and the figure design and approved the review for publication. All authors contributed to the article and approved the submitted version.

## Conflict of Interest

The authors declare that the research was conducted in the absence of any commercial or financial relationships that could be construed as a potential conflict of interest.

## Publisher’s Note

All claims expressed in this article are solely those of the authors and do not necessarily represent those of their affiliated organizations, or those of the publisher, the editors and the reviewers. Any product that may be evaluated in this article, or claim that may be made by its manufacturer, is not guaranteed or endorsed by the publisher.
